# Phasic and sustained interactions of multisensory interplay and temporal expectation

**DOI:** 10.1038/s41598-018-28495-7

**Published:** 2018-07-05

**Authors:** Felix Ball, Fabienne Fuehrmann, Fenja Stratil, Toemme Noesselt

**Affiliations:** 1 Biological Psychology, Faculty of Natural Science, Otto-von-Guericke-University Magdeburg, Magdeburg, Germany; 2 Center for Behavioural Brain Sciences, Otto-von-Guericke-University Magdeburg, Magdeburg, Germany

## Abstract

Every moment organisms are confronted with complex streams of information which they use to generate a reliable mental model of the world. There is converging evidence for several optimization mechanisms instrumental in integrating (or segregating) incoming information; among them are multisensory interplay (MSI) and temporal expectation (TE). Both mechanisms can account for enhanced perceptual sensitivity and are well studied in isolation; how these two mechanisms interact is currently less well-known. Here, we tested in a series of four psychophysical experiments for TE effects in uni- and multisensory contexts with different levels of modality-related and spatial uncertainty. We found that TE enhanced perceptual sensitivity for the multisensory relative to the best unisensory condition (i.e. multisensory facilitation according to the max-criterion). In the latter TE effects even vanished if stimulus-related spatial uncertainty was increased. Accordingly, computational modelling indicated that TE, modality-related and spatial uncertainty predict multisensory facilitation. Finally, the analysis of stimulus history revealed that matching expectation at trial n-1 selectively improves multisensory performance irrespective of stimulus-related uncertainty. Together, our results indicate that benefits of multisensory stimulation are enhanced by TE especially in noisy environments, which allows for more robust information extraction to boost performance on both short and sustained time ranges.

## Introduction

Optimisation of perceptual processing is critical in daily life. Misperceiving or even missing targets in our environment can have severe consequences (e.g. in traffic). Thus, how perceptual processing is facilitated has been a central tenet in psychology for decades. Several mechanisms governing perceptual facilitation have been identified, among them spatial attention^1–8^, object- and feature-based attention^9–11^, temporal expectations (TE)/attention^12–22^ and multisensory interplay (MSI)^23–34^. All these mechanisms have in common that target-related performance (e.g. detection or discrimination of targets) is enhanced when targets occur e.g. at attended locations, possess certain features, are presented at expected moments in time (instead of unexpected moments), or when targets are multisensory (instead of unisensory). Additionally, some studies investigated synergistic effects between the aforementioned mechanisms. For instance, there is corroborating evidence for the interactions of spatial attention with MSI^35^ and with temporal attention^21^. However, the interplay of TE and MSI has not been thoroughly investigated.

Previous studies on crossmodal temporal processing had often focused on the transfer of knowledge of temporal regularities across modalities. For instance, in Lange *et al*.^36^ participants attended either short or long target-intervals, and either the auditory or tactile modality. The authors reported that response times were speeded for expected targets in the attended - but also in the unattended modality. This indicates that subjects possess the ability to transfer knowledge (here TE) from one modality to the other^36,37^, though other studies provided evidence to the contrary^20,38^. Beyond the question of whether TE acts on a modality-specific or supramodal level, there are only few studies testing the influence of multisensory stimulation - and in particular of audiovisual stimulation - on TE. So far, most investigations on TE rather utilized unisensory stimuli instead (e.g. only visual or auditory)^39^ and investigated e.g. how iterative Bayesian modelling of temporal processing may account for changes in unisensory processing^22^. However, many real-life events stimulate more than one sensory modality in isolation. Thus, utilizing multisensory stimulation may reveal critical insights into TE-related psychological mechanisms in more ecologically valid situations.

A recent multisensory study on the effects of TE^20^ had reported enhanced TE effects in multisensory (audiovisual [AV]) relative to unisensory contexts (auditory [A] and visual [V])^20^. The goals of that previous report were to introduce a new paradigm to investigate TE and to test how robust group-mean TE effects were across experimental conditions and experiments. However, that analysis approach did not take into account subject-specific preferences which are critical when identifying multisensory facilitation (MSF, i.e. multisensory vs. best unisensory response, see below). Imagine the following: one participant prefers and attends only one modality, say auditory. Then, performance in the auditory condition could be 100% while in the visual condition it could be 50% (i.e. at guess rate). Attending only the auditory modality in the combined multisensory condition would most likely also result in 100% accuracy but may not reflect MSF. The second participant might prefer the visual modality and so on. Therefore, this approach might be less sensitive to the identification of MSF. Importantly, at the level of group-analysis preferred and non-preferred modalities would be averaged across participants in our previous study, obfuscating potential effects of MSF.

Here, we will focus on *subject-specific* multisensory interactions instead and address the question whether the interaction of TE and MSI results in ‘true’ multisensory facilitation. To this end, we will base our analysis on the multisensory max-criterion^40^ rather than comparing accuracies condition-wise. The max-criterion posits that multisensory performance should exceed the best unisensory performance (maximum perceptual sensitivity or minimum response time), if participants use sensory information of both modalities (auditory and visual). Hence, the max-criterion allows to quantify subject-specific MSF relative to unisensory performances in the preferred modality^40,41^ (in contrast to our previous report which focuses on condition-specific comparisons). Note that with MSI, we will refer to a more general, underlying mechanism of stimulus processing, while MSF refers to the (behavioural) read-out as computed by “audiovisual vs. best unisensory condition”.

To investigate interactions of MSI and TE, classical univariate analyses can be used to determine mean performance differences across conditions. Other modelling approaches^33,42–44^ such as cross-validation allow to complement results and to test whether a model trained on data X can be used to predict independent data Y. If data Y can be successfully predicted, the parameters of the model may reflect the underlying cognitive process. Using this approach, we addressed the critical question whether MSF is best described (predicted) by physical factors (such as proximity of the stimuli, target frequencies etc.) and/or by individual factors (such as the preferred unisensory modality). Note that especially a preference for one modality might strongly impact MSI/MSF. On the neural level, audiovisual facilitation is less likely when neurons exhibit a response imbalance to unisensory stimulation^41^. It remains to be seen how this neural response pattern might be transposable to the behavioural level during TE. Potentially, the preference for one modality might decrease the likelihood of properly processing the non-preferred modality. Hence, MSF might be reduced compared to the case when both modalities are equally processed. Here, we will use a complementary cross-validation modelling approach to determine the factors governing MSI in a TE paradigm.

Finally, we will investigate the effects of phasic short-term adaptation of TE and MSI^22,45–51^, as recent reports had indicated that phasic–in addition to sustained effects–may also play a role in MSI^45–47^. Moreover, there are some studies on the foreperiod paradigms indicating that trial-by-trial history can alter temporal attention. In foreperiod studies, the participant is primed on each trial that a stimulus appears after a certain delay. Whenever a trial has a short foreperiod and is preceded by trials with longer foreperiod, reaction times are increased^48–51^, indicating that short-term effects may indeed modulate TE^22^. Hence, we will test whether matches in temporal target position (TP) of the previous trials (at n-1) differently affect performance in the current trial for uni- and multisensory trials. Together, these analyses should provide a detailed picture of MSI, TE, and their interaction.

In four experiments, participants judged the frequency of uni- or multisensory targets embedded in a stream of distractors. Time of occurrence (early vs. late) of targets was manipulated block-wise^52^. Moreover, reliability of target modality (knowing vs. not knowing target’s modality) and spatial position (speaker vs. headphones) was manipulated across experiments to identify the situations in which TE and MSI effects occur and interact. Based on the previous findings on TE, we hypothesised (1) that perceptual sensitivity should increase and response times should decrease whenever targets are expected in time^39^. Most importantly, if MSI and enhancement by TE interact, we should observe (2) larger TE effects in the multisensory condition^20^. This facilitation should be stronger (3) when uncertainty about target’s location and identity increase^20,30–32^. Concordantly with these hypotheses, (4) the best fitting computational model should include the factors TE, spatial and target uncertainty. Moreover, (5) enhanced preference for one modality should decrease MSF. Finally, (6) participants’ performance should increase if trials are preceded by a match in temporal target position^48–51^. Given that multisensory stimulation showed higher sensitivity for temporal information^20^, (7) short-term TP effects should be enhanced in multisensory trials.

## Methods

The results presented in this paper are based on a re-analysis of data collected in 4 experiments which were already presented in earlier work^20^. Note that the previous analyses did neither focus on MSI nor phasic effects of temporal expectancy and MSI. For reader’s convenience, we list the relevant information again. Please be referred to our previous work^20^ for further details (i.e. use of different reaction time selection criteria, participant exclusion criteria, etc.).

### Participants

Here we analysed data of 120 participants tested in a series of four experiments (30 participants each). All participants provided written informed consent and declared to be free of neurological or psychiatric disorders and to have normal or corrected visual acuity. The demographic data is listed in Table 1. This study was approved by the local ethics committee of the Otto-von-Guericke University, Magdeburg. The methods were carried out *in accordance with* the relevant guidelines and regulations.

**Table 1:** Information about participants. Mean age +/− standard deviation of the 30 participants per experiment, their sex and handedness are listed for each experiment.

Experiment	Age	Women	Left-handed
Exp1	24.5 ± 2.7	13	2
Exp2	23.1 ± 3.4	18	0
Exp3	24.3 ± 3.6	21	4
Exp4	23.9 ± 3.7	22	2

### Apparatus and stimuli

Psychophysics Toolbox (Version 3)^53^, Matlab 2012b (Mathworks Inc.) and a LCD screen (22”, 120 Hz, SAMSUNG 2233RZ) recommended for vision research^54^ were used for stimulus presentation. Speakers (Piezo Super Tweeter, Conrad Electronics, Germany; Experiments 1 and 3) or headphones (Sennheiser HD 650; Experiments 2 and 4) were used for acoustic stimulation. Monitor resolution was set to 1650 × 1080 pixels and the refresh rate to 60 Hz. The viewing distance was 102 cm (eyes to fixation point). A wireless mouse (Logitech M325) was used as response device.

We used chequerboards (3.07°) and/or pure sinusoidal sounds (ramp of 5 ms) for uni- and multisensory stimulation. Stimuli were always presented in a sequence of 11 events (100 ms stimulus, 100 ms gap). Chequerboards were presented 2.31° above the (white) fixation cross (which itself was presented 2.9° above the screen’s centre). Stimulus background was dark grey (RGB: 25.5). If speakers were used, sounds were presented 3.22° above chequerboard’s upper edge (yet vertically aligned).

Targets and distractors within the stimulus sequence differed in frequency. Visual (4.6, 4.9, and 5.2 cycles per degree) and auditory (2975, 3000, and 3025 Hz) distractor frequencies were randomized. Target frequencies (auditory and visual) were adjusted to 75% accuracy level at the beginning of the experiment (mean target frequencies were virtually identical across experiments^20^).

### Procedure

**Figure 1:**
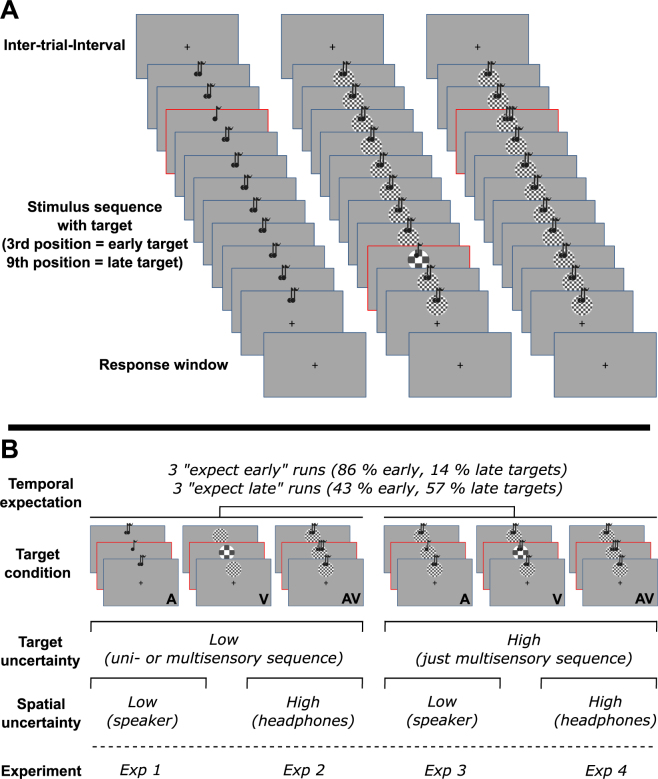
Experimental Design. (**A**) Three exemplary stimulus sequences (left: auditory-only, middle: multisensory with redundant target, right: multisensory with just auditory target). Each trial started with a blank screen (inter-trial-interval) followed by a sequence of 11 auditory (Exp 1 + 2), visual (Exp 1 + 2), or audiovisual stimuli (all Exp). Stimuli were presented for 100 ms with a 100 ms gap in between. The stimulus sequence ended with a blank screen (response window, max. 1500 ms). Targets were either presented at the 3rd or 9th position. Note that squares highlight the target (lower or higher frequency than distractor items) for illustrative purposes only and were not present in the experiment. In Experiments 3 and 4 only multisensory sequences were used (middle and left sequence). (**B**) Overview of factorial experimental design. Depicted are all 6 possible target conditions and factor levels of spatial and target uncertainty. The experiment’s number (bottom) denotes in which experiment a particular combination of factor levels was used. Note that manipulations of temporal expectations were identical across experiments.

All four experiments had in common that they consisted of one training block, two threshold determination blocks, 3 “expect early” and 3 “expect late” blocks (as described below). Each experiment was conducted in a dark, sound-attenuated chamber. Each trial consisted of a sequence of 11 stimuli (100 ms) which were separated by a 100 ms gap. Sequences were either auditory, visual, or combined audiovisual. In Experiments 1 and 2, we used unimodal auditory, unimodal visual and audiovisual sequences with unimodal auditory, visual and audiovisual targets. Audiovisual targets were always redundant in frequency. In Experiments 3 and 4, sequences were always audiovisual, but targets were – as in Experiments 1 and 2 – either just auditory, just visual or redundant audiovisual. Auditory and visual streams in the multisensory condition were always presented synchronously. There was exactly one visual, auditory or audiovisual target stimulus per sequence, presented at the 3^rd^ (400 ms after sequence onset) or 9^th^ position (1600 ms after sequence onset). Participants were required to identify the deviant stimulus in the sequence and to judge its frequency (higher or lower than remaining distractor stimuli) as fast and accurately as possible. Participants used both thumbs for response input (response- thumb mapping was counterbalanced across participants). Responses were collected from the start of the sequence until 1500 ms after the sequences ended. The inter-trial-interval (200–400 ms) was initiated by button press or the end of the 1500 ms interval (see Fig. 1 for design).

Before the start of the experimental session, participants completed one training block (24 trials) and two threshold-determination blocks (144 trials). Here, we used only auditory and visual sequences with an equal number of early and late targets. The threshold-determination blocks were used to adjust target’s frequency to 75% accuracy level. In the main experiment (1008 trials total, 168 trials per block, 56 trials per target modality [A, V, AV]), we presented two types of blocks to manipulate temporal expectations. In one block (“expect early”), the early target was more likely (86% early targets, 14% late targets). In the other block (“expect late”), the late target was more likely (43% early targets, 57% late targets). There were three “expect early” and three “expect late” blocks in each experiment, and the starting block type was counterbalanced across participants. Participants were naïve about the temporal manipulation but knew about target presence in each trial.

Differences between experiments were created by manipulating the context in which stimuli were presented. Thereby, we manipulated participants’ certainty about the spatial position and modality of the upcoming target stimulus. In Experiments 1 and 3, we used speakers in close proximity to the visual stimulus. Hence, stimuli were always presented in front and were thereby always spatially predictable (low spatial uncertainty). In Experiments 2 and 4, auditory stimuli were presented via headphones, thereby rendering the spatial position of the stimulus sequence unpredictable across trials (frontal screen and/or headphone; high spatial uncertainty). Hence, some participants had to only focus on one spatial position in the low spatial uncertainty experiments, while other participants had to dynamically shift their focus on a trial by trial basis in the high spatial uncertainty experiments. Target-specific modality uncertainty was kept low in Experiments 1 and 2. In these experiments, targets were embedded in unisensory or multisensory stimulus sequences. Hence, the sequence type (A, V, or AV) automatically determined the upcoming target. In Experiments 3 and 4, we used only multisensory sequences but presented unisensory or multisensory targets (e.g. only the auditory stimulus had a higher/lower frequency than distractors). Thus, participants had to monitor both stimulus streams to perceive the unisensory targets (high target uncertainty).

### Analysis

As in our previous report, only early target trials were included within a response window ranging from 150 to 3000 ms after target onset^20^ (see Supplemental material V for a complementary analysis of late target performance). To quantify TE benefits, we compared expected and unexpected early targets. Late target performance was not analysed further as late targets are always expected and may thus not require temporal attention (in line with findings in many studies^20,36,39,52,55–57^). As in our previous study^20^, two different performance measures were calculated. We used the perceptual sensitivity index dʹ for two-alternative forced choice (2AFC) tasks^58^ and mean response times (RTs). Matlab 2012b (Mathworks Inc.), R (version 3.4.0 with *ezANOVA* package version 4.4.0) and JASP (v. 0.8.4.0) were used for statistical analysis.

To test for MSF, we compared the best unisensory performance (auditory or visual) to performance in the multisensory condition^40,59^. The best unisensory modality was determined in a maximum two steps: either we chose the modality with best performance in both sub-conditions (temporally expected vs. unexpected) or – in case of a tie (e.g. A for expected and V for unexpected) – the one showing the best average performance. Note that modality was allowed to differ across performance measures (maximum dʹ and minimum RT) for each participant. In each experiment, a maximum of 8 participants showed different best modalities for the two measures (see 3.1). From now on, we will refer to the best unisensory modality with ‘best [A,V]’ and to the audiovisual condition with ‘AV’.

To test for an interaction of multisensory enhancement and enhancement by TE (hypotheses 1–3), maximal unisensory and multisensory performance scores (RT and dʹ) were subjected to a repeated-measures ANOVA with within subject factors *modality* (best[A,V], AV) and *TE* (expected, unexpected). Furthermore, we added between subject factors *spatial uncertainty* (low, high) and *target uncertainty* (low, high) to test for possible influences of uncertainty on performance. As effect size index, we used generalized ƞ^2^ (ƞG^2^) and ƞ^2^ as computed by the *ezANOVA* package and JASP. Post-hoc tests were Bonferroni-corrected (p_Bonf_) to account for multiple comparisons and Bayes factors in favour of H0 (BF_H0_) were reported for all non-significant p-values.

### Modelling

To test which factors optimally predict the label of MSF for each participant (hypotheses 4–5), we conducted a modelling analysis. To this end, we modelled our data on the basis of a generalized linear regression using the glmfit function in Matlab^3^. Analyses were run separately for dʹ and RT scores. The to-predicted values were the difference of “AV – max[A,V]” for dʹ, and “min[A,V] – AV” for RT scores. This way positive values always indicated multisensory facilitation and negative values always indicated no facilitation. The prediction parameters were split into three groups. The first were the physical parameters: spatial uncertainty (low, high), target uncertainty (low, high), temporal regularities (probable vs. improbable target positions), normalized auditory target frequency, and normalized visual target frequency (frequencies were normalized with their respective mean across all experiments). The second set of parameters comprised individual data: best unisensory modality as determined by both performance measures (auditory, visual, or mixed; see beginning of Results section). The third and last set of parameters comprised unisensory performance data. The reason for including this parameter derived from the idea that just like neurons^41^ (or maybe as a by-product of neural activity), participants might have a strong preference for one modality and thereby, ignore the other modality. To this end, we determined the difference of “max[A,V] - min[A,V]” for dʹ, and “min[A,V] - max[A,V]” for RT scores, respectively.

These differences were subjected to individual models. We computed as many models as there were combinations of prediction variables (8 predictors, amounting to a total of 255 models). The data models had a total of 240 data points (N) of the to-predicted values. The validity of the model fits was evaluated using a leave-one-out cross validation procedure. In particular, the data points were divided into a training set of N-1 data points and a single test data point. The linear regression model was computed for the set of training data points, and its weights were used to predict the response of the test data point. This procedure was repeated 240 times, such that each data point served as test data once, allowing us to compute the difference between predicted and observed dʹ and RT scores of each data point. The success of each model was then quantified by computing the models’ root mean square error (RMSE; difference between predicted and observed values). Additionally, we computed Spearman correlations (rho) between estimated and observed data for the best model, the physical parameters model, the individual data model, the unisensory performance data model, and the full model. We further computed the variance explained by each predictor of the best models as measured by the Proportionate Reduction of Error (PRE)^60^.

### Analysis of trial history

In addition to sustained effects of TE and MSI, phasic effects of stimulus history may also influence ongoing behaviour. To investigate effects of the immediate past (preceding trial) on the current trial (hypotheses 6-7), we focused on dʹ scores. Our previous report^20^ as well as the univariate and the modelling approaches in this paper conclusively showed that dʹ was the more sensitive measure of modality-specific effects (i.e. performance AV > best[A,V]) in the present series of experiments. For completeness, results of sequential effects for RTs are reported in the supplementary material (Supplementary III).

In our analysis, we calculated dʹ scores for the n trial under the restriction that the n-1 trial was either a modality match (e.g. V trial preceded by V trial) or a mismatch (e.g. V trial preceded by A trial). This factor is labelled *modality match*. We also calculated dʹ scores for the n trial under the restriction that the n-1 trial was either a target position (TP) match (e.g. unexpected trial preceded by unexpected trial) or a mismatch (e.g. expected trial preceded by unexpected trial). This factor is called *TP match*. We calculated scores for all combinations of these factors for AV and best[A,V] trials (factor *modality*). Note that we controlled for other factors in the n-1 trial to avoid their influence on the n trial. To include the n trial into analyses, the answer in the n-1 trial had to be correct, thereby avoiding post-error influences. To analyse dʹ scores, we used an ANOVA with within subject factors *modality, modality match* and *TP match*. Furthermore, we added between subject factors *spatial uncertainty* (low, high) and *target uncertainty* (low, high) to test for possible influences of uncertainty on performance.

### Data availability statement

The datasets generated during and/or analysed during the current study are available from Open Science Framework^61^. All remaining information are available from the corresponding author upon request.

## Results

### Descriptive statistics: Modality preference/maximal unisensory performance

Across all experiments, a higher number of participants showed enhanced performance in the unisensory auditory relative to the visual condition (total number of participants: 56 auditory [47%], 36 visual [30%]). For 28 participant (23%) the modality with highest performance was different for the two performance measures (dʹ vs. RT, see Table 2 for details). Based on the response measure, a total of 69 [58% for dʹ] or 71 [59% for RT] participants showed enhanced performance in the auditory condition.

**Table 2:** Subject-specific modality preferences per experiment.

Experiment	Auditory	Visual	Mixed	A preference	
				dʹ	RT
Exp1	14	10	6	18	16
Exp2	12	10	8	15	17
Exp3	16	8	6	18	20
Exp4	14	8	8	18	18

Number of participants with maximal performance (dʹ and RT) in the unisensory auditory condition, in the unisensory visual condition, or mixed preferences for dʹ and RT across experiments (N_total_ = 120). Last two columns list how many participants preferred the auditory modality depending on dʹ or RT.

### Inferential statistics: MSI is strengthened by TE under high uncertainty

First, we tested whether the patterns of dʹ and RT scores are indicative of an enhancement by TE or MSI, an interaction of MSI and TE and whether any effect is further modulated by spatial or modality-specific target uncertainty. Perceptual sensitivity was enhanced and response times were shortened for expected compared to unexpected targets (main effect of TE: dʹ of 1.272 and 1.089, respectively; F(1,116) = 45.912, p < 0.001, ƞG^2^ = 0.034, ƞ^2^ = 0.277; RTs of 1523.6 and 1634.96 ms, respectively; F(1,116) = 73.087, p < 0.001, ƞG2 = 0.017, ƞ^2^ = 0.378), in accord with our hypothesis. The same was true for the audiovisual compared to best unisensory condition (main effect of modality: dʹ of 1.24 and 1.122, respectively; F(1,116) = 15.431, p < 0.001, ƞG2 = 0.015, ƞ^2^ = 0.098; RTs of 1549.8 and 1608.8 ms, respectively; F(1,116) = 31.898, p < 0.001, ƞG^2^ = 4.7*10^−3^, ƞ^2^ = 0.172), again as hypothesised. Furthermore, perceptual sensitivity was decreased in the high compared to low target uncertainty experiments (main effect of target uncertainty: dʹ of 1.073 and 1.289, respectively; F(1,116) = 7.678, p = 0.007, ƞG^2^ = 0.047, ƞ^2^ = 0.062) while RTs were unaffected (F(1,116) = 0.012, p = 0.915, BF_H0_ = 1.9).

For both dʹ and RTs the main effect of modality was further modulated by target uncertainty (modality × target uncertainty: dʹ - F(1,116) = 20.815, p < 0.001, ƞG^2^ = 0.02, ƞ^2^ = 0.132; RT - F(1,116) = 37.118, p < 0.001, ƞG^2^ = 0.5.5*10^−3^, ƞ^2^ = 0.2). For dʹ the interaction was driven by a significant decrease of performance in the best unisensory condition under high compared to low uncertainty (0.945 and 1.298, respectively; T(118) = 4.574, p_Bonf_ < 0.001, d = 0.835), while performance was not significantly different in the multisensory condition (10.202 and 1.279, respectively; T(118) = 0.872, p_Bonf_ = 0.77, BF_H0_ = 3.7). For RTs, there was no difference between high and low uncertainty in the best unisensory and multisensory condition (t(118) < = 0.904, p_Bonf_ > = 0.736, BF_H0_ > = 3.5). However, MSF differed depending on the uncertainty level. While there was no MSF under low target uncertainty (AV = 1586 ms, best[A,V] = 1581 ms, t(59) = 0.311, p_Bonf_ = 1, BF_H0_ = 6.8), RTs were facilitated for audiovisual stimuli under high target uncertainty (AV = 1514 ms, best[A,V] = 1636 ms, t(59) = 8.503, p_Bonf_ < 0.001, d = 1.1). Perceptual sensitivity was further modulated by spatial uncertainty (modality × spatial uncertainty: F(1,116) = 4.474, p = 0.037, ƞG^2^ = 4.4*10^−3^, ƞ^2^ = 0.028). While d′ did not differ between uni- and multisensory trials under low uncertainty (1.218 and 1.163, respectively; T(59) = 1.19, p_Bonf_ = 0.478, BF_H0_ = 3.6), it decreased stronger in the best unisensory condition under high spatial uncertainty (1.263 and 1.08, respectively; T(59) = 3.933, p_Bonf_ < 0.001, d = 0.508). This two-way interaction was non-significant for RTs (F(1,116) = 0.03, p = 0.862, BF_H0_ = 6.5).

**Figure 2:**
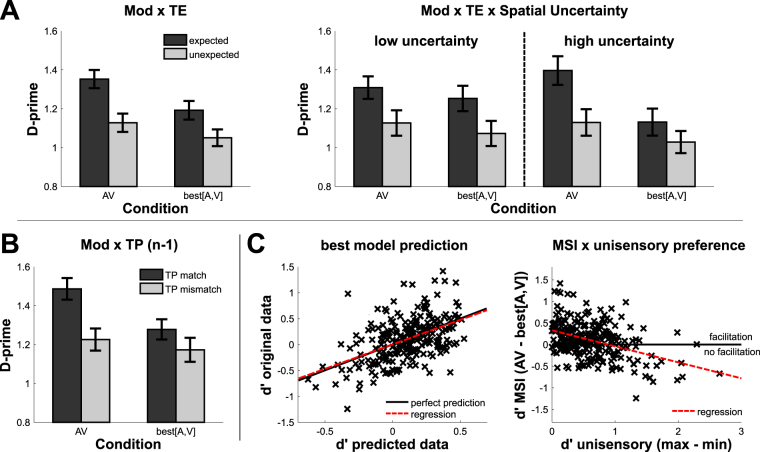
Inferential and modelling results. (**A**) Mean dʹ values (+/−SE) of the significant interactions “modality × TE” and “modality × TE × spatial uncertainty”. dʹ values are displayed for the audiovisual (AV) vs. best unisensory conditions (best[A,V]). Expected trials are displayed in dark grey, unexpected trials in light grey. (**B**) Mean dʹ values for sequential effect (n – 1) interactions of “modality × TP”. Target position (TP) match trials are displayed in dark grey, TP mismatch trials in light grey. (**C**) Results of the d′ modelling analysis. Left: Best model prediction of individual data points (x). The black line depicts a perfect prediction and the red dotted line the regression of all data points. Right: Interaction of MSI with unisensory preference. Red dotted line is regression of all data points. Y-values above the black indicate multisensory facilitation (AV > best[A,V]). Large X-values indicate that participants strongly preferred one modality (max[A,V]≫min[A,V]).

The critical comparison pertains to the question whether the results would reveal an interplay of TE and MSI. Indeed, we found a significant interaction of both factors (modality × TE: F(1,116) = 4.246, p = 0.042, ƞG^2^ = 1.8*10^−3^, ƞ^2^ = 0.034; see Fig. 2A). Enhancement of perceptual sensitivity by TE (expected - unexpected) was larger for audiovisual than for the best unisensory condition (dʹ difference of 0.225 and 0.141, respectively; T(119) = 2.05, p = 0.043, d = 0.187, Fig. 2A). Moreover, this interaction was further modulated by spatial uncertainty (modality × TE × spatial uncertainty: F(1,116) = 4.058, p = 0.046, ƞG^2^ = 1.8*10^−3^, ƞ^2^ = 0.033, Fig. 2A). Under low spatial uncertainty, TE (expected - unexpected) effects did not differ between the best unisensory and multisensory conditions (dʹ difference of 0.182 and 0.18, respectively; T(59) = 0.034, p_Bonf_ = 1, BF_H0_ = 7.1). However, under high spatial uncertainty the TE effects were larger in the multisensory condition (dʹ difference of 0.103 and 0.267, respectively; T(59) = 2.824, p_Bonf_ = 0.001, d = 0.365), due to a non-significant TE effect in the best unisensory condition (best[A,V]: T(59) = 1.761, p_Bonf_ = 0.167, BF_H0_ = 1.7; AV: T(59) = 5.98, p_Bonf_ < 0.001). All remaining dʹ and RT effects were non-significant (dʹ: F(1,116) < = 2.295, p > = 0.133, 6.7 > = BF_H0_ > = 2.1; RT: F(1,116) < = 3.104, p > = 0.081, 5.3 > = BF_H0_ > = 1.7 except for interaction TE × target uncertainty: BF_H0_ = 0.7 and interaction spatial × target uncertainty: BF_H0_ = 1.2). For results of late targets, see Supplement V.

### Factors predicting labels of multisensory facilitation

A mixture of physical and performance parameters optimally predicted multisensory enhancement as measured by dʹ (RMSE: 0.36, rho = 0.5, p_Bonf_ < 0.001; see Fig. 2C). These are spatial uncertainty, target uncertainty, temporal regularity and unisensory-dʹ-difference. In particular, multisensory enhancement increased with increasing spatial and target uncertainty and when targets were unexpected. Furthermore, MSF decreased when the unisensory-dʹ-difference became larger (see Fig. 2C). Thus, all factors which had an effect in the inferential statistics were also included in the best model, but the best fitting model also included the additional predictor unisensory preference. Note that target properties included in the full model did not further enhance model fit.

For the best RT model, we found a similar result (RMSE: 122.14, rho = 0.52, p_Bonf_ < 0.001). Here, MSF increased with high target uncertainty and decreased when visual targets became more distinct from distractors and when the unisensory-RT-difference became larger. For completeness, we list the RMSE and correlation results of the best (here mixed parameters) model, the 3 different parameter set models, and the full model (comprising all sets of parameters) in Table 3 (see Supplement I for an illustration of beta estimates across models). Further, we list the beta estimates of the best models in Table 4.

**Table 3:** Results model analysis. dʹ models are presented in the upper part and RT models in the lower part.

Measure	Model	Rank	RMSE	Rho	p_Bonf_
dʹ	Best	1	0.361	0.501	<0.001
	Physical	138	0.393	0.332	<0.001
	Individual	245	0.418	−0.022	1
	Uni Perf	105	0.384	0.388	<0.001
	Full	22	0.363	0.491	<0.001
RT	Best	1	122.14	0.523	<0.001
	Physical	97	128.32	0.446	<0.001
	Individual	213	143.293	0.057	1
	Uni Perf	180	139.573	0.228	0.002
	Full	40	123.734	0.506	<0.001

Listed are the rank (out of 255 models, 1 indicating best model), the RMSE, the Spearman correlation (rho) and its p-value (pBonf). Abbreviations used: Best = best fitting model, Physical = physical parameters model, Individual = individual data model, Uni Perf = unisensory performance data model, Full = full model, RMSE = root mean square error.

**Table 4:** Beta estimates of the best model fits. Betas are shown for the two performance measures (dʹ upper part, RT lower part) and each factor.

Measure	Factor	Beta	P	SE	PRE
dʹ	intercept	−0.054	0.676	0.13	—
	SpU	0.089	0.055	0.046	0.016
	TaU	0.248	<0.001	0.046	0.11
	TE	0.092	0.048	0.046	0.017
	dʹ unisensory	−0.343	<0.001	0.05	0.166
RT	intercept	−94.296	<0.001	26.83	—
	TaU	127.647	<0.001	15.67	0.22
	Freq (V)	−23.848	0.003	7.97	0.037
	RT unisensory	−0.173	<0.001	0.037	0.083

Further, we list the p-value, standard error (SE) and the Proportionate Reduction of Error (PRE, i.e. the variance explained) for each beta. SpU = spatial uncertainty, TaU = target uncertainty, TE = temporal expectation (expected, unexpected), dʹ /RT unisensory = unisensory difference (best – worst) for the respective measure, Freq (V) – normalized visual frequency.

### Influence of n-1 trials on performance in uni- and multisensory conditions

Finally, we investigated whether short-term, phasic modulations based on previous target position (TP) or modality presentation (n trial vs. n-1 trial) could affect task performance in addition to the sustained effects reported above. As we focus on effects of trial history, we only report main effects and interactions which include the relevant factors *modality match* and *TP match*. As expected, perceptual sensitivity increased when the previous trial was a modality match (dʹ of 1.372 and 1.21, respectively; F(1,116) = 21.183, p < 0.001, ƞG^2^ = 0.013, ƞ^2^ = 0.15) or a TP match (dʹ of 1.382 and 1.199, respectively; F(1,116) = 17.893, p < 0.001, ƞG^2^ = 0.017, ƞ^2^ = 0.131). More importantly, there was also a difference between best [A,V] and AV for dʹ scores (modality × TP match: F(1,116) = 4.406, p = 0.038, ƞG^2^ = 3.1 ∗ 10^−3^, ƞ^2^ = 0.035; see Figure 2B): a match of TP between the current and previous trial resulted in increased perceptual sensitivity in the multisensory (T(119) = 4.8, p_Bonf_ < 0.001, d = 0.438) but not in the best unisensory condition (T(119) = 1.755, p_Bonf_ = 0.164, BF_H0_ = 2.23). There was a tendency that this effect is further modulated by spatial uncertainty (F(1,116) = 3.746, p = 0.055, BF_H0_ = 1.2; see Supplement II). All remaining interactions for dʹ were non-significant (F(1,116) < = 3.154, p > = 0.08, 9.9 > = BF_H0_ > = 1.8). Response times were again less sensitive to capture the difference between the uni- and multisensory conditions as all significant response time effects did not include factor modality (see Supplement III and IV for RT results and further discussion of effect sizes).

## Discussion

In this study, we investigated whether temporal expectation (TE) and multisensory interplay (MSI) are two in- or interdependent mechanisms facilitating behavioural performance. Critically, we tested the time-scale on which these mechanisms interact and whether any effects reflect MSI using the max-criterion, computational modelling, and trial-history analyses.

We found that participants’ perceptual sensitivity was increased and response times were decreased for expected compared to unexpected trials, and for multisensory compared to the best unisensory trials, in accord with our hypotheses. Remarkably, TE and MSI interacted; i.e. facilitation by TE was nonlinearly enhanced in the multisensory condition. In accord, computational modelling revealed that multisensory facilitation (MSF) is best described by a mix of physical parameters (temporal regularities, spatial and target uncertainty) and, most importantly, the differences of best minus worst unisensory performance. We also observed that performance was influenced on a trial-by-trial basis in addition to the sustained TE and MSI effects. Overall, performance was increased whenever trials were preceded by a trial which matched in modality or target position. Importantly, the target position match with the previous trials specifically enhanced multisensory performance irrespective of stimulus-specific uncertainty. Together, our results support the notion that TE and MSI – which have been previously studied in isolation – are cooperative mechanisms that interactively strengthen target feature perception.

The sustained effects observed here extend previous reports on RT-related TE effects by adding modulations of perceptual sensitivity and changes due to uni- vs. multisensory contexts. Most notably, we also found phasic trial-history effects on perceptual sensitivity which differed for uni- vs. multisensory conditions. Previous research on trial-history effects focussed either on unisensory temporal expectations^48–51^ or on MSI^45–47^. In the TE studies^48–51^, reaction times decreased whenever short foreperiod trials (time between cue and target) were preceded by matching short foreperiod trials, while RTs increased when the preceding trials had a mismatching long foreperiod. These findings suggested that processing the temporal structure of the current trial will modulate response readiness for the upcoming trial. The multisensory studies^45–47^ had shown that e.g. physical synchrony of audio-visual stimulus pairs can influence synchrony judgements in the upcoming trial^47^. Furthermore, audiovisual spatial mismatch can affect mismatch perception (i.e. ventriloquist effect) in the upcoming trial^45,46^. Our study adds to these previous unisensory, phasic and sustained TE results^12–19,39^ that in addition to increases in response readiness, sensory representations (as indicated by dʹ effects) can be enhanced. Furthermore, by investigating uni- and multisensory sustained and phasic TE effects we were able to show that phasic as well as sustained TE effects differentially affect uni- and multisensory processing.

On the sustained level, we observed that the modality effect (i.e. AV > best[A,V]) was only significant for expected trials. On the trial-by-trial level, matches in temporal target position (TP) enhanced multisensory perceptual sensitivity regardless of the modality of the preceding trial. Potentially, the processing of target’s temporal position sets a temporal position marker for the next trial. However, this short-term temporal position marker is only effectively boosting perception if multisensory stimulation occurs (i.e. larger TE effects occur irrespective of spatial and target uncertainty). Mechanistically, the increased perception of multisensory targets might be caused by a lowered response criterion or a speeded evidence accumulation due to short-term TE^62^. In contrast, unisensory evidence accumulation – especially under high uncertainty – might be insufficient to exceed the response threshold or is insufficiently accelerated by TE. Hence, TE most likely boosts MSI and thereby AV target contrast but less so unisensory target contrast.

While the mechanism proposed above suggests that TE influences MSI, the interaction of TE and MSI might be also partly determined by the influence of MSI on TE. Under high spatial uncertainty, a situation which had not been introduced in most previous TE experiments – sustained TE effects vanished in the best unisensory condition but not the multisensory condition (see also^20^ for individual A and V effects). A likely explanation for this pattern of results would be the following: In our experiments, participants were oblivious about the manipulation of TE (as by instruction and corroborated by reports of post-hoc questioning^20^). Thus, they had to rely on their perception of the target, and had to extract the statistical distribution of early and late target trials within each block to direct their attention accordingly. If unisensory targets were simply less often perceived (i.e. more targets were missed which is supported by the dʹ results), it may have been more difficult to create statistics about unisensory target occurrence. In contrast, multisensory events were perceived more often in general and especially under high uncertainty conditions. Hence, summary statistics could thus be more easily obtained for multisensory stimulation but are apparently not fully transferred between modalities^20,38^.

Thus, the mechanisms governing phasic and sustained effects of TE would start with short-term TE which is generated supramodally. This supramodal generation we observed would also be in line with other studies showing that in rhythmic streams and within the same trial, expectations about the timing of the upcoming stimulus within a sequence are created supramodally^36,37^. Short-term TE interacts with MSI, thereby rendering a better estimate about the cumulative statistics of temporal target occurrence. This cumulated estimate is equivalent to the sustained TE effects observed here and driven by the multisensory context. It remains to be seen in future studies, whether the cognitive plasticity (as indexed by phasic TE effects) is maximal at the beginning of each block and gradually decreases over trials when more evidence about temporal trial structures becomes available and temporal rules are extracted (the number of trials was too low in our experiments and prevents an empirical test of this hypothesis at this point). Further, it needs to be tested whether an increase in trial number would have led to a robust extraction of temporal rules in the unisensory target trials or whether the extraction of temporal structure for one target modality actively prohibits additional rule extractions for other modalities.

When directly comparing performance in unisensory target conditions, we found more incidents of higher performance in the auditory (~60% of subjects) compared to the visual condition (~40%). One might argue that the performance imbalance is indicative of a threshold procedure problem. This reasoning would certainly be true for standard multisensory experiments without a manipulation of temporal regularities^25,34^. However, the threshold procedure prior to the main experiment adjusted for each participant both unisensory conditions to a 75% performance level under the restriction that temporal attention is not manipulated (i.e. same number of early and late target trials were presented). Ultimately, the manipulation of temporal attention in the main experiment affects both unisensory conditions, and targets in expected trials will be judged correctly more often. If one modality has a superior temporal precision, it should support the creation of TE more strongly. Indeed, several studies have pointed at the auditory dominance in the temporal domain which is likely due to its higher temporal resolution^39,63,64^. Remarkably, most of the previous literature on TE effects is based on the visual domain while this domain seems to be the least susceptible to temporal regularities. Future TE studies should incorporate different modalities to classify modality-specific TE difference within the same participants and paradigms.

In addition to classical univariate analysis, we used a leave-one-out modelling approach to identify which factors are predictive of performance. Given our model approach data, MSF was best described by a mixture of physical parameters (dʹ model: spatial and target uncertainty, TE; RT model: target uncertainty and visual target frequency) and participants’ performance in the unisensory conditions (dʹ model: unisensory-dʹ-difference; RT model: unisensory-RT-difference). The larger the performance difference between the unisensory conditions, the less multisensory enhancement occurred. Hence, participant-specific modality preference determined behavioural MSF in close resemblance to the (im)balances of neural firing rates determining audiovisual neural facilitation in anesthetized cats^41^. The preference predictor also explained most of the variance (best fitting dʹ-model: 1^st^ rank; best fitting RT-model: 2^nd^ rank), further emphasizing its importance. Importantly, stronger modality preferences seem to reduce (or even prohibit) integration and/or the use of both information streams. The preference for one modality might be determined by the importance of each modality for the task at hand (e.g. spatial vs. temporal task). Another factor might be the experimental context and participants’ strategy. It seems that most participants relied more on a single modality in easier tasks (i.e. low uncertainty experiments)^20^. Most likely, they tried to optimize their overall performance as our experiment still applied a complex task with different modalities, distracting stimuli etc. Hence, attending to only one modality may have reduced attentional switch costs (switching in space [headphone experiments] and between modalities across trials) and potentially increased the chance to identify targets occurring in the preferred modality. Such strategy would optimize performance on two thirds of the trials (multisensory and favourite modality trials) but decrease the chance of MSF.

Moreover, spatial and target uncertainty seem to differentially influence MSI as measured by dʹ and RTs (as indexed by both the univariate as well as leave-one-out approach). MSF based on dʹ is affected by both types of uncertainty which suggests that both types decrease unisensory target visibility and thereby higher the chance that MSI can enhance performance by boosting stimulus contrast. Note that participants put accuracy before speed in our paradigm^20^. Hence, no evidence for a violation of the race model^59^ was observed which is typically used to test for MSI effects based on reaction times. Here, we only found mean differences in response times which were driven by modality-specific uncertainty and visual contrast. The effect of modality-specific uncertainty may be best explained by the higher informational content of AV stimuli which are more easily detectable when participants do not know about the modality of the upcoming target. The effect of visual contrast is less straightforward but might again be explained by participants’ preference. As more participants preferred the auditory modality, MSF was dependent on visual target visibility. When visual target-distractor differences were small, participants only relied on the auditory stimulus thereby decreasing the incidents of MSF. When the difference was large, this visual information was taken into account (possibly integrated) to enhance performance. In sum, our results suggest that MSI is driven by an interplay of several different factors. However, our data also suggest that average RT effects might reflect decision processes rather than perceptual latency/facilitation. Note that to confirm their initial percept, participants tended to respond at the end of the sequence instead of directly after the presentation of the target^20^. There were only a few cases with response times below 1000 ms. Further, RTs correlated negatively with accuracy (R = 0.4); the less accurate participants were (i.e. the more difficulty they had to judge target’s frequency) the slower they responded. Hence, fast responses were only given when participants were certain about target’s frequency or when they guessed unexpected late unisensory targets (see Supplement V). Thus, like in other experiments^65^ choice response times in the current study most likely captured post-perceptual processing – determined by participants’ response criterion and motor preparation – rather than representing solely perceptual processing.

Although target frequencies were virtually identical across experiments^20^, high target uncertainty decreased unisensory performance and increase the incidents of MSF. Thus, it appears that stimulus effectiveness (e.g. their individually perceived salience) was not solely determined by physical factors but rather by internal factors – i.e. how well stimuli are processed and thereby their salience is enhanced. One could term this phenomenon ‘internal effectiveness’. Whenever targets are embedded among distractors and non-targets their internal effectiveness is lowered and participants are more uncertain about their answer. In turn, presenting a multisensory, redundant stimulus diminishes distraction by providing higher (doubled) informational content. This might increase the chance that information is treated as important and used (integrated) to solve the task. This would be an addition of the classical 3^rd^ rule of multisensory integration (“inverse effectiveness”^30–32,41^) which only makes assumption about the physical (external) effectiveness of stimuli (e.g. the lower the contrast the higher the chance of MSF) and which is mostly studied in simple detection tasks. However, in complex environments with suprathreshold stimuli, internal effectiveness might be the more relevant parameter. Studies showed e.g. that the movement of an approaching, suprathreshold stimulus can be more properly judged when this stimulus is paired with a sound^29^. Thus, MSI/MSF as determined by internal effectiveness might be especially important in e.g. traffic, when suprathreshold stimuli can be easily missed (as shown by e.g. change blindness experiments^3–5^). To summarize, while physical effectiveness can determine MSF in simple paradigms, internal and physical effectiveness might determine MSF in more complex paradigms.

In conclusion, our results revealed that MSI and temporal attention cooperatively enhance perception. This benefit occurs not only on a sustained level, but can be observed on a trial-by-trial basis. Remarkably, the stimulus position of the previous trial was only informative when followed by multisensory target trials but occurred independently of the modality of the previous trial. This suggests that information about the temporal structure cannot be assessed as readily in unisensory contexts or does not enhance unisensory target processing as much as multisensory target processing. For the latter, TE might increase the chance of MSI which appears to increase target saliency and thus, identification. Multisensory facilitation is also determined by participants’ preference in unisensory processing. The more participants focus one modality, the less they use and combine audiovisual information. This imbalance between modalities might partly be driven by the task’s complexity. Together, our results indicate that temporal expectations predominantly enhance multisensory perception (in discrimination paradigms) by potentially boosting MSI on both short and sustained time ranges.

## Electronic supplementary material

Supplementary material
