# Supplementary material for: Phasic and sustained interactions of multisensory interplay and temporal expectation
Source: Sci Rep. 2018 Jul 5;8:10208. doi: 10.1038/s41598-018-28495-7 (PMC6033875; doi:10.1038/s41598-018-28495-7)
Supplement: Supplementary file 1 — Supplementary material [file 41598_2018_28495_MOESM1_ESM.pdf]

Supplementary material

Phasic and sustained interactions of multisensory interplay and temporal expectation

Felix Ball[1,2*], Fabienne Fuehrmann[1], Fenja Stratil[1], Toemme Noesselt[1,2]

[1] Biological Psychology, Faculty of Natural Science, Otto-von-Guericke-University Magdeburg, Germany
[2] Center for Behavioural Brain Sciences, Otto-von-Guericke-University Magdeburg, Germany

Supplement I: Beta estimates for all models

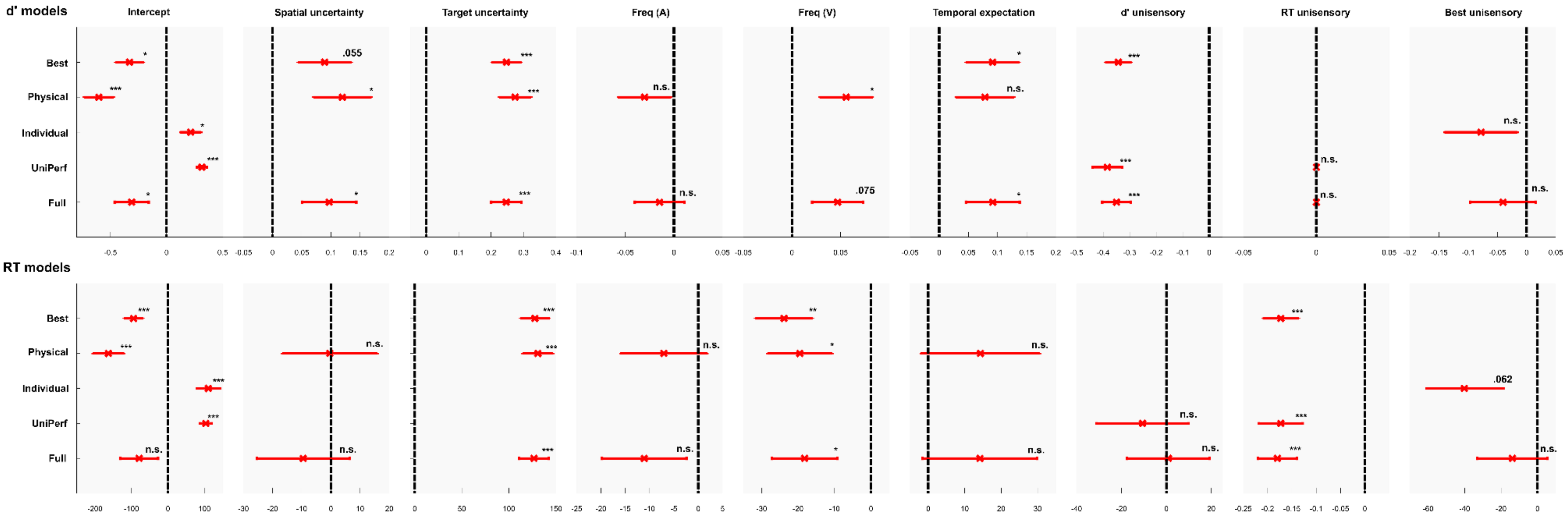

Supplementary Figure 1: Here we show all beta estimates for the d' models (top) and the RT models (bottom) reported in our publication. Beta estimates are shown for each factor (columns) as well as the intercept. Error bars depict standard errors. Significance values are indicated as follows: *** p<.001, ** p<.01, * p<.05, n.s. implies p>.1.

Abbreviations used: Best = Best fitting model, Physical = physical parameters model, Individual = individual data model, Uni Perf = unisensory performance data model, Full = full model, Freq (A) = normalized auditory frequency, Freq (V) = normalized visual frequency, d' /RT unisensory = unisensory difference (best – worst) for the respective measure, Best unisensory = best unisensory condition (A, V, or mixed).

Supplement II: d-prime “n-1 analysis”

As mentioned in the main text, there was a tendency that the interaction of modality and TP match is further modulated by spatial uncertainty ($F(1,116) = 3.746$, $p = .055$, $BF_{H0} = 1.2$). Similarly to the sustained effects, TE effects (here target position) did not differ under low spatial uncertainty between AV and best[A,V] conditions (d' difference of .264 and .2, respectively; $T(59) = .606$, $p_{Bonf} = 1$, $BF_{H0} = 5.9$). Under high uncertainty, TE effects differed (d' difference of .276 and .047, respectively; $T(59) = 2.399$, $p_{Bonf} = .04$, $d = .31$) and this effect was driven by the absence of the TE effect in the best unisensory condition (best[A,V]: $T(59) = .556$, $p_{Bonf} = 1$, $BF_{H0} = 6.1$; AV: $T(59) = 3.237$, $p_{Bonf} = .004$).

Supplement III: RT sequential effects

Main effect modality match:

RT_{Match}=1627 ms, $RT_{mismatch}$=1653 ms; $F(1,116)=12.928$, $p<.001$, $\eta G^2 = 9.1*10^{-4}$, $\eta^2 = .093$

Main effect target position match:

RT_{Match}=1560 ms, $RT_{mismatch}$= 1720; $F(1,116)=122.451$, $p<.001$, $\eta G^2 = .004$, $\eta^2 = .511$

Interaction modality match x target uncertainty:

$F(1,116)=7.844$, $p=.006$, $\eta G^2 = 5.5*10^{-4}$, $\eta^2 = .057$

**mean RT in ms**	Modality Match n-1	Modality Mismatch n-1
Low uncertainty	1611.1	1656.2
High uncertainty	1643.6	1649.2

Supplementary Table 1: Summary - High target uncertainty generally increased RTs. Under high uncertainty modality match did not modulate RTs. Under low uncertainty, modality match slightly decreased RTs compared to modality mismatch.

Interaction modality match x target position (TP) match:

$F(1,116)=10.628$, $p=.001$, $\eta G^2 = 8.1*10^{-4}$, $\eta^2 = .082$

**mean RT in ms**	Modality Match n-1	Modality Mismatch n-1
TP match n-1	1534.99	1584.3
TP mismatch n-1	1719.7	1721.2

Supplementary Table 2: Summary - TP mismatch generally increased RTs. Modality match was only relevant when TP matches with previous trial. A combined match slightly decreased RTs.

All remaining interactions

$F(1,116)<=3.475$, $p>=.065$, $9.8 >= BF_{H0} >= 2.5$

Supplement IV: Discussion of effect sizes

It should be noted that effect sizes for the significant interactions (TE*Mod, TE*Mod*spatial uncertainty) are relatively small. Larger effect sizes were restricted to the highly significant main effects.

However, we did not expect that the interaction of TE and modality would result in a large effect size. A large interaction effect size would imply that almost all participants show multisensory enhancement and a larger enhancement in the expect condition *across all experiments*. Given the manipulation of experimental context (i.e. spatial and target uncertainty) across experiments this is highly unlikely. For example, in the low target and low spatial uncertainty experiment (Exp.1), task difficulty was minimised compared to all other experiments. Hence, multisensory stimulation might be less beneficial. In accord with this notion, 14 out of 30 participants consistently did not show multisensory facilitation there. Furthermore, 7 participants in this experiment showed multisensory enhancement only in the unexpected condition, i.e. the more difficult condition due to missing preparedness. In all 4 experiments combined, 24 of 120 participants showed no sign of multisensory facilitation and 16 participants showed no improvement due to TE (4 people did neither show TE nor multisensory enhancement). For a total of 15 out of 120 participants, multisensory enhancement was restricted to unexpected trials. However, the majority of 81 participants showed multisensory facilitation in the expected condition especially in the high spatial uncertainty experiments which gave rise to the reported interaction effect size.

Based on our previous report, we could have restricted our analysis to those experiments with a robust overall interaction (i.e. experiments with high spatial uncertainty) as this interaction is the main focus of our manuscript. A reduced ANOVA approach would have indeed resulted in a highly significant 'TE*modality' interactions with a pointedly larger effect size (i.e. $\eta^2 = .12$). However, we chose – in the interest of the readership – to report the full scope of our results, thereby reducing effect sizes.

Finally, effect sizes might have also been affected by the ratio of early and late target trials in the 'expect early' and 'expect late' blocks (86%-14% and 43%-57%, respectively). The reason behind our decision to use more early trials in the expect-late blocks was to have a more robust estimate of unexpected early trials. Performance in the expected early condition is based on 144 trials and in the unexpected early condition on 72 trials. If we would fully reverse probabilities, unexpected early performance would be based on 24 trials which significantly lowers reliability of the performance measure. However, it most likely would have increased the overall TE effect (see Exp. 5 [1]) and could have also affected the interaction term.

Supplement V: Late targets

As for early targets, the auditory modality was the preferred modality when targets were presented late (d': 72 of 120, RT: 66 of 120).

Perceptual sensitivity for late targets was largely unaffected. Performance was still improved when targets were audiovisual (modality: $F(1,115) = 8.322$, $p = .005$, $\eta G^2 = .011$, $\eta^2 = .066$) and when target uncertainty was low ($F(1,115) = 11.183$, $p = .001$, $\eta G^2 = .005$, $\eta^2 = .087$).

There was also a trend that these 2 effects interacted ($F(1,115) = 2.912$, $p = .091$, $BF_{H0} = 1.3$). All other effects were non-significant (including the TE effect; all $F(1,115) \leq 2.63$, $p \geq .108$, $7.2 \geq BF_{H0} \geq 1.6$).

For RTs, we found a significant interaction of modality*target uncertainty ($F(1,115)=4.572$, $p=.035$, $\eta G^2 = .001$, $\eta^2 =.038$) and modality*TE ($F(1,115)=12.894$, $p<.001$, $\eta G^2 = .003$, $\eta^2 = .1$). The graphs below illustrate that under high uncertainty, RTs increased in the best unisensory condition (Supplementary Supplementary Figure 2 left), and RTs decreased when unisensory targets were unexpected (Supplementary Supplementary Figure 2 right). All other effects were non-significant (including the TE effect; all $F(1,115) \leq 1.08$, $p \geq .3$, $8.5 \geq BF_{H0} \geq 1.5$).

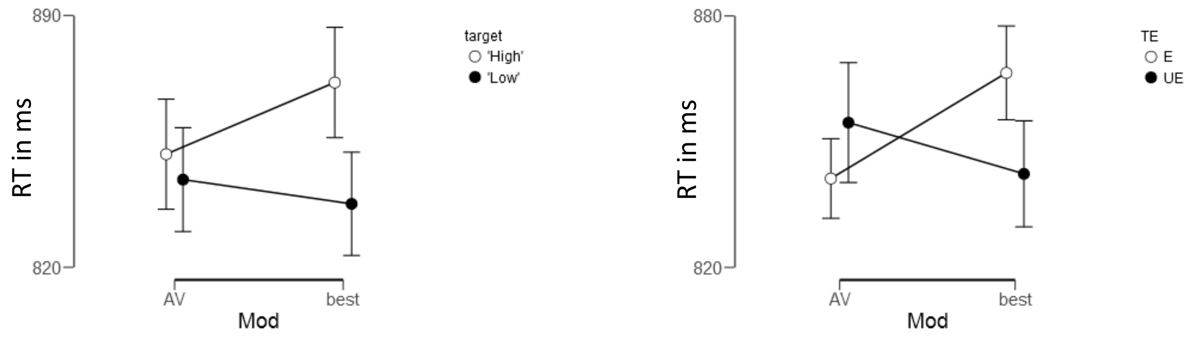

Supplementary Figure 2: RT plots for late targets. Left: Interaction of modality and target uncertainty. Right: interaction of modality and TE. Error bars are 95% confidence intervals. Abbreviations: AV = audiovisual, best = best unisensory condition, Mod = modality, target = target uncertainty, TE = temporal expectation (E = expected, UE = unexpected).

As in our previous report[1], the late target results support the notion that late targets are always expected and that TE effects are restricted to scenarios with temporal uncertainty. Yet audiovisual stimulation can still enhance target perception (as indicated by the d' effect). However, the pattern of results found for RTs rather indicates that differences were driven by decision processes. If e.g. perceptual latency was truly shortened, we would expect that reaction times decrease in the expected condition (as for AV trials, see Supplementary Figure 2 right). However, here RTs decreased in the unexpected condition indicating that participants possibly lowered their response threshold in the unexpected unisensory condition. This condition was most likely less often perceived than the audiovisual condition, hence, participants guessed more often fast than slow.

References

1. Ball, F., Michels, L. E., Thiele, C. & Noesselt, T. The role of multisensory interplay in enabling temporal expectations. *Cognition* **170,** 130–146 (2018).
